# Reducing Stigma, Enhancing Psychological Well-Being and Identity in Multiple Sclerosis: A Narrative Review of Current Practices and Future Directions

**DOI:** 10.3390/healthcare13111291

**Published:** 2025-05-29

**Authors:** Cristina Montesano, Giulia Liberali, Gianluca Azzali, Cosme Buzzachera, Sonia Angilletta, Marco Alessandria, Laura Guidetti, Andrea De Giorgio

**Affiliations:** 1 Department of Theoretical and Applied Sciences, eCampus University, 22060 Novedrate, Italy; sonia.angilletta@uniecampus.it (S.A.); marco.alessandria@uniecampus.it (M.A.); andrea.degiorgio@uniecampus.it (A.D.G.); 2 Department of Public Health, Experimental and Forensic Medicine, University of Pavia, 27100 Pavia, Italy; giulia.liberali@unipv.it (G.L.); cosme.buzzachera@unipv.it (C.B.); 3 Department of Humanities, Movement, and Education Sciences, University “Niccolò Cusano”, 00166 Rome, Italy; azzali.dott.chinesi@gmail.com (G.A.); laura.guidetti@unicusano.it (L.G.)

**Keywords:** social withdrawal, illness narratives, self-compassion, self-coherence, peer support

## Abstract

Background: Stigma is a pervasive, though understudied, psychosocial factor affecting people with multiple sclerosis. This review synthesizes the literature on the impact of perceived, enacted, and internalized stigma on psychological health and illness identity in PwMS. Methods: A comprehensive narrative review approach was adopted, integrating findings from peer-reviewed quantitative and qualitative studies. Databases including PubMed, PsycINFO, and Scopus were searched using combinations of terms such as “multiple sclerosis”, “stigma”, “internalized stigma”, “mental health”, and “illness identity”. Studies were included if they reported on stigma-related outcomes in PwMS, discussed psychological or identity variables, or examined interventions aimed at reducing stigma or enhancing adaptive identity. The analysis focused on thematic synthesis, identifying recurrent findings, mediating mechanisms, and clinical implications. Results: Stigma in MS is consistently linked to higher depression, anxiety, and lower quality of life. Internalized stigma disrupts illness identity, often fostering engulfment and rejection of the self. Psychological mediators—such as cognitive fusion, diminished self-compassion, and weakened sense of coherence—amplify these outcomes. Particularly vulnerable are individuals with progressive disease, severe disability, minority status, or limited social support. A recursive loop emerges: stigma triggers psychological distress, which increases stigma awareness and social withdrawal. In addition to traditional psychological interventions, several emerging approaches have shown promise in reducing internalized stigma and fostering adaptive identity integration. Conclusions: Stigma is a central factor in emotional suffering and identity fragmentation in PwMS. Integrative, narrative-informed, and culturally sensitive interventions are essential to reduce stigma and foster resilience. Future research should prioritize longitudinal, mixed-method approaches to develop effective, scalable solutions.

## 1. Introduction

Stigmatization is a widespread social process with a profound impact on the daily lives of people living with chronic illness. In 2001, the World Health Organization defined stigma as “A sign of shame, disgrace or disapproval that causes a person to be rejected, discriminated against and excluded from participation in various spheres of society”. The American Psychological Association (2018) further describes stigma as a socially mediated process that exacerbates psychological burden, whereby people are discredited or devalued based on real or perceived characteristics. As such, stigma affects people’s psychosocial adjustment in multiple and multidimensional ways. Beyond interpersonal prejudice, stigma also operates through systemic mechanisms that support inequality and marginalization [1,2]. Although the stigma associated with HIV has been widely investigated [3], the role of stigma in multiple sclerosis (MS) remains relatively unexplored. A growing body of evidence, however, points to its significant impact on mental health and self-concept [4,5]. MS is a chronic, immune-mediated disorder of the central nervous system, characterized by unpredictable motor and cognitive impairments. Societal stereotypes, concerns about visibility-related insecurities, and the inherent unpredictability of the disease, make people with multiple sclerosis (PwMS) particularly vulnerable to stigmatizing experiences [6,7,8].

Recent research has begun to specify the nature of manifestations of stigma in the MS population. Importantly, the literature distinguishes between perceived stigma, which refers to the awareness or expectation of negative societal judgments, internalized stigma, in which people absorb and accept such judgments, and practiced stigma, denoting overt discriminatory behaviors [9,10]. Internalized stigma is particularly harmful, as it contributes to shame, self-blame, and avoidance behaviors, while impairing help-seeking and well-being [11]. Anticipated stigma, in turn, has also been shown to predict social withdrawal, which in turn negatively affects mental health and community adjustment, particularly in individuals with covert stigmas such as criminal records [12]. Although stigma is rarely the primary focus in MS literature, studies using stigma-specific indices consistently associate it with adverse psychological outcomes. Excessively elevated levels of anxiety, depression, and low self-esteem have been reported in individuals who perceive or internalize stigma [13,14,15,16]. Stigma has also been linked to suicidality in PwMS, further underscoring its clinical relevance.

Importantly, stigma in MS affects not only mood, but also illness identity—a key psychological construct describing how individuals categorize and make sense of the illness in relation to their self-concept [17]. Internalized stigma may disrupt autonomy and psychological coherence, leading to a state of engulfment, where the illness overwhelms the personal self [18,19]. On the other hand, psychosocial moderators such as resilience, social support, and self-compassion can mitigate these effects and facilitate healthier identity schemas. Overall, the experience of stigma in MS intersects significantly with the core domains of mental health and identity. Understanding this interplay is critical to developing individualized interventions that move beyond MS-related symptom management to more fully address the psychological and existential dimensions of living with MS.

This review aims to summarize the current literature on stigma in MS, with a particular emphasis on its effects on psychological well-being and illness identity, the mediating mechanisms involved, and clinical implications that emerge from the current data.

## 2. Stigma and Psychological Well-Being in MS

Stigmatization is recognized as a powerful psychosocial stressor that significantly affects the psychological well-being of PwMS [2]. In addition to the physical and neurological impact of the disease on the whole body, PwMS often experience considerable psychological disturbances such as depression and anxiety, which, in turn, diminish quality of life. These challenges are often exacerbated by perceived, acted out and internalized forms of stigma. Empirical evidence consistently shows a robust association between stigma and higher levels of both depression and anxiety, and reduced quality of life in PwMS. A large-scale study using Neuro-QoL and PROMIS assessment tools found that stigma had a significant negative impact on physical and mental health in PwMS, with part of this impact mediated by mood-related symptoms, such as depression and anxiety [20]. More recently, a systematic review further highlighted the cumulative effect of anticipated, experienced, and internalized stigma in MS, concluding that these forms of stigma are strongly associated with impaired psychological functioning and reduced health-related quality of life [21]. These findings are confirmed by studies focusing on individuals with primary progressive MS, indicating that stigma remains highly prevalent and independently predicts depressive symptoms and poor mental health, even in subpopulations with distinct disease trajectories [22]. Other efforts have sought to explore the psychological mechanisms that may mediate the relationship between stigma and mental health. For example, cognitive fusion—enmeshment with negative thoughts—has been shown to mediate the effects of stigma on depression, anxiety and quality of life in PwMS. Therefore, psychological interventions could help strengthen psychological resilience [23] as the literature highlights that stigma is not a peripheral phenomenon, but a central determinant of psychological outcomes. Hence, addressing stigma through routine clinical screening, targeted therapeutic interventions, and structural change is crucial for improving quality of life and supporting mental health in this population.

### 2.1. The Emotional Cost of Being Stigmatized

There is substantial evidence indicating that both experienced and internalized stigma are significantly associated with depressive and anxiety symptoms among PwMS. For example, a cross-sectional survey of the MS-PATHS cohort found that 17.7% of PwMS reported moderate to severe levels of stigma, with higher stigma scores predicting poorer outcomes on the neurological subscales of the Neuro-QoL tool [20]. Similarly, PwMS with elevated levels of internalized stigma were significantly correlated with higher levels of depression and anxiety, reduced social support, and diminished willingness to seek treatment [9]. Similar findings were reported in the *Judgment Hurts* study by Cadden et al. [2], which demonstrated that stigma independently predicted emotional distress, particularly depressive symptoms, even after adjusting for disease severity and sociodemographic factors. The relationship between stigma and psychological distress is, therefore, complex. Internalizing stigma, wherein individuals adopt harmful illness beliefs about their condition, foster feelings of self-diminishment, shame, and hopelessness [24]. Anticipated stigma—the fear of future judgment—has been shown to encourage concealment, social withdrawal, and hypervigilance, which, in turn, reduces access to social support, a critical protective factor against depression and anxiety [25]. In addition, stigmatization, especially when it occurs within healthcare or workplace contexts, can amplify feelings of invisibility and powerlessness [26]. Such experiences intensify exacerbation by heightening fears of devaluation and marginalization [27]. Overall, the existing evidence underscores the notion that stigma is not merely a social nuisance, but a clinically significant moderator of mental health in PwMS. Addressing its effects requires a comprehensive, multifaceted psychosocial approach.

### 2.2. Mediators and Moderators of Psychological Outcomes

There is increasing evidence that the relationship between stigma and mental health in MS is mediated by psychological variables such as cognitive fusion, self-compassion, sense of coherence and rumination [23,28,29]. These variables influence how people internalize and respond to stigma, rendering them either more vulnerable to its psychological consequences or, conversely, more resilient. For example, cognitive fusion—the tendency to identify with negative self-descriptive thoughts—has been shown to mediate the association between internalized stigma and depressive symptoms in PwMS. In a study by Valvano et al. [23], cognitive fusion significantly mediated the relationship between MS-related stigma and symptoms of depression and anxiety, suggesting that individuals who were more readily convinced by the negative thoughts triggered by stigma are more prone to suffer from stress. This is in line with findings from other chronic illness populations, which suggest that psychological inflexibility amplifies the psychological impact of stigma on mental health [30]. Another key resilience factor is the sense of coherence, i.e., the perception that the world, including oneself, is understandable, manageable and meaningful. A strong sense of coherence has been found to buffer the negative effects of stigma on depression in patients with chronic illness, representing a protective mindset that promotes stability and adaptation [31]. Self-compassion also emerges as a protective factor associated with reduced vulnerability to the psychological consequences of stigma. In a psychometric evaluation of the Persian version of the cognitive fusion questionnaire in PwMS, self-compassion was negatively correlated with both cognitive fusion and depressive symptoms [32]. Practicing a self-compassionate stance towards personal suffering may mitigate internalized stigma and enhance emotional resilience. Social support remains one of the most robust protective factors. Individuals who report strong emotional and social connections experience significantly fewer negative psychological effects from stigma [21]. Peer support groups and group-based interventions can offer the psychological benefit of emotional recognition as well as the added benefit of a shared identity that can combat shame and social isolation. These observations are further substantiated by Powell et al. [33], who conducted a comprehensive systematic review examining the association between stigma and health outcomes in PwMS. The authors collected data from over 22,000 participants across 18 studies, reporting a consistent and significant link between perceived stigma and a broad spectrum of adverse psychological and physical health outcomes. For example, two longitudinal studies, cited by Powell and collaborators in the review, highlight that stigma at baseline predicted clinical depression at one-year follow-up, suggesting not only a correlational but potentially causal relationship. Moreover, the role of psychological processes—such as cognitive fusion and sense of coherence— has been shown to mediate the impact of stigma, thus pointing to actionable targets for intervention. The findings also emphasized the role of demographic and social variables, including race, employment status, and disease visibility, in shaping stigma experiences and health vulnerabilities. Powell et al. [33] advocated for the implementation of evidence-based psychological interventions—particularly cognitive–behavioral and mindfulness-based approaches—that have demonstrated efficacy in reducing stigma and improving psychological well-being in PwMS. Their model positions stigma not merely as a social or attitudinal issue, but as a modifiable psychosocial determinant of health with tangible effects on disease trajectory and patient outcomes [33]. Collectively, these findings emphasize that the psychological effects of stigma are modifiable. Psychological concepts such as cognitive fusion, sense of coherence, and self-compassion, along with access to supportive social networks, offer concrete interventions for the psychological burden of stigma in MS.

### 2.3. Vulnerability Factors: Who Is Most at Risk?

Numerous studies have identified specific subgroups within the population of PwMS who are particularly vulnerable to the psychological effects of stigma. Individuals with progressive forms of MS, greater physical disability, lower educational attainment and unemployment are particularly vulnerable. A large cross-sectional analysis of the MS-PATHS cohort found that individuals with progressive MS and higher disability scores were significantly more likely to report experiencing stigma, which in turn was associated with poorer quality of life and mental health outcomes [34]. Progressive MS is often characterized by more visible impairments and a scarcity of effective disease-modifying treatments, contributing to a greater sense of devaluation and marginalization. In a targeted study of individuals with primary progressive MS, 78.2% reported experiencing stigma, which independently predicted depression symptoms and reduced quality of life [22]. Gender differences have also been observed, with women with MS more likely to report stigma than men, a disparity that may reflect societal expectations around caregiving roles, appearance, and productivity [35]. Moreover, individuals with MS identifying as LGBTQ+ may experience increased stigma due to the intersection of chronic illness with minority stress. Anderson et al. [36] emphasized the ways in which sexual and gender minority individuals struggle with double concealment—either hiding their MS in LGBTQ+ communities or hiding their orientation/sexuality in MS groups—leading to greater alienation and psychological distress. Employment status also plays a significant role. The loss of work due to MS can be both a consequence and a source of stigmatization and psychological distress. Unemployed PwMS tend to report higher levels of stigma and lower psychological quality of life. A Canadian survey found that feelings of stigma were strongly associated with unemployment and greater unmet needs for informal care, even after controlling for disability severity and sociodemographic factors [37]. Environments with inadequate provisions and environments that subtly exclude people with chronic health conditions can also contribute to feelings of worthlessness that exacerbate depression and anxiety. Taken together, these findings underscore the need for both targeted psychological interventions and broader structural reforms to better support the most vulnerable subgroups within the MS population in managing the effects of stigma.

### 2.4. The Stigma–Well-Being Feedback Loop

Stigma not only contributes to psychological distress but can also be perpetuated by it, thereby creating a self-sustaining cycle. Depression and anxiety, common consequences of internalized and perceived stigma in PwMS, can disrupt social functioning, diminish engagement in daily activities, and impair self-care. These consequences often lead to concealment and withdrawal, which in turn reinforce the experience and internalization of stigma. This vicious cycle describes how stigma and mental disorders are mutually reinforcing, highlighting the importance of early psychological intervention aimed at breaking this dynamic. The process of internalizing stigma is underpinned by identifiable cognitive-affective mechanisms such as, for example, the cognitive fusion [23]. Because of this, individuals become entangled with negative self-evaluative thoughts (e.g., “I am defective because I have multiple sclerosis”), leading to emotional distress and behavioral avoidance [38]. Another central process is anticipated stigma, which involves the expectation of rejection or discrimination, fostering hypervigilance and biased interpretation of ambiguous social cues as stigmatizing [39]. According to Pinel’s model of stigma awareness, members of marginalized groups become hypersensitive to signs of prejudice, which exacerbates avoidance behaviors, intensifies emotional suffering, and further re-establishes social disconnection [40,41]. The increased sensitivity can lead to a rise in emotional upset, social withdrawal, and chronic stress responses [40,42]. Furthermore, the self-discrepancy theory can further provide insight [43]. Indeed, when individuals perceive a discrepancy between their actual self and the standards of normalcy or competence valued by society, this can lead to the formation of internal conflict and self-derogation, and thus heighten stigma-related distress. These interconnected processes imply that internalized stigma is not only a social process but a psychologically mediated process influenced by maladaptive meaning, attentional bias, and experiential avoidance. It is here that psychological therapies prove their worth and not merely as tools for symptom relief, but as targeted modulators of the cognitive–emotional architecture that supports stigma. The literature data emphasize the therapeutic potential of interventions that not only alleviate suffering but also interrupt self-perpetuating cycles of stigma and mental health impairment [44,45]. Such treatments may be particularly critical for PwMS who contend with challenges related to bodily appearance, identity, and perceived social judgment, requiring both clinical and psychosocial support.

### 2.5. Implications for Clinical Care

Recognizing the impact of stigma on mental health in PwMS is crucial for delivering holistic, person-centered care [46,47]. Routine mental health screening should explicitly assess the experience of stigma, particularly in individuals exhibiting signs of social withdrawal, demoralization, or reluctance to engage with treatment. Targeted interventions that support self-compassion, enhance cognitive plasticity and foster peer affiliation have demonstrated particular efficacy. For instance, mindful self-compassion training has been shown to reduce self-criticism and psychological distress [48], while simultaneously enhancing resilience and social acceptance in populations with chronical illness and among marginalized groups [49,50]. These interventions are effective both in traditional in-person formats and virtual delivery, thereby improving accessibility and scalability. Peer support represents another vital pillar. Programs that engage individuals with lived experience—either as peer educators or group facilitators—are especially effective in fostering social connection, facilizing identity reconstruction, and mitigating stigmatization, including within the context of clinical education [51,52]. Clinician education plays an equally crucial role. Training programs that involve direct interaction with people who have lived experience and that address implicit bias and social distancing head-on have been shown to significantly reduce stigmatizing attitudes among clinicians. These training programs also improve diagnostic competency and building greater trust in the clinician–patient relationship [53]. Ultimately, adopting a trauma-informed, stigma-sensitive care model that validates lived experiences, promotes empowerment, and recognizes the multifaceted nature of stigma is essential. Such approaches not only enhance psychological well-being, but also preserve dignity, support identity restoration, and foster resilience throughout the course of illness.

## 3. Stigma and Illness Identity in Multiple Sclerosis

Illness identity refers to the ways individuals accept, reject or negotiate their disease as part of their personality [17,54]. Although psychological health and illness identity are depicted as distinct outcomes, they are closely related and can exert a bidirectional complex influence on each other. Literature suggests that stigma-induced disruptions in illness identity (for instance, increased engulfment or rejection) can serve as mediating mechanisms through which stigma has a detrimental impact on psychological health. For example, Stepleman et al. [18] confirmed that identity types characterized by distress and lack of integration were associated with higher psychological burden in PwMS. Similarly, Tang and Lin [19] illustrated how resilience played a mediating role in the relationship of stigma on illness identity, and it affected emotional outcomes. Such findings indicate that illness identity is not solely an outcome of stigma but also a psychological pathway linking stigma to general emotional distress. Therefore, the consideration of the bidirectional influence between identity and mental health is fundamental for obtaining a full picture of the psychosocial burden of stigma in PwMS. In the context of MS, the interplay between stigma and illness identity plays a significant role. The internalization, resistance, or reframing of stigmatizing experiences profoundly impacts how people make meaning, agency and continuity in the world. Qualitative research shows that PwMS often have difficulty integrating the illness into their self-structure without being overwhelmed by it. In a landmark case study, Riessman [55] explored how men with MS gripped with masculine identity, bringing together idealized societal notions of strength with the lived truth of limited abilities due to the disease. Narratives serve not only to make sense of disruptive experiences but also to reclaim agency and social belonging. Stepleman et al. [18] developed a validated framework for reconceptualizing identity in MS, identifying three primary identity types: maintained identity (a stable sense of self), reactionary identity (characterized by resistance and distress), and integrated identity (an inclusive self that reconciles both pre-and post-illness self). Notably, integrated and maintained identities were associated with more adaptive psychological functioning and lower levels of stigma [18]. Stigma, however, not only threatens identity, but can also affect identity recovery mechanisms. Grytten and Måseide [27] observed that PwMS often perceive themselves as “sicker” in social interactions due to the reactions they receive from others, which heighten self-consciousness and reinforce an illness-oriented identity. Such disruption of attempts to present a coherent self-occur when these self-presentation efforts are reinforced, reinforcing the illness-oriented identity and self-stigma of the PwMS [27].

### 3.1. Theoretical Perspectives on Illness Identity

Illness identity has been conceptualized in different ways within the literature, but it consistently refers to how individuals understand, relate to, and integrate a chronic illness into sense of self. One of the foundational models is Charmaz’s [56] theory, which introduces the notion of “loss of self”—a perceived threat to identity when agency and continuity are undermined. This disruption is particularly pronounced in conditions such as MS, in which uncertainty and stigma further challenge self-perception. More recently, Oris et al. [54] developed the Illness Identity Questionnaire, which delineates four key orientations toward illness identity: engulfment, rejection, acceptance, and enrichment. These dimensions represent a continuum of adjustment. Engulfment occurs when the illness consumes the individual’s self-perception. Rejection expresses a denial of the illness as part of the self. Acceptance, in turn, entails incorporating the illness, where the self is not defined by the illness. Finally, enrichment describes self-growth and transformation through the illness [17]. Stigma, especially when internalized, has a direct and detrimental influence on the development of a coherent and adaptive illness identity. Individuals exposed to high levels of perceived or experienced stigma are more prone to developing an engulfing illness identity, where the illness becomes a dominant, negative component of self-definition. Engulfment is associated with higher depression, lower self-esteem, and lower quality of life in a number of chronic illnesses, including MS [19]. In contrast, protective psychosocial resources such as social support and resilience can buffer the effects of stigma and enable the development of more adaptive illness narratives, such as acceptance and enrichment. For example, research shows that resilience serves as a mediator in the relationship between stigma and illness identity—reducing the likelihood of engulfment and increasing the likelihood of developing enriching or accepting illness narratives [19]. Therefore, the development of acceptance and enrichment could alleviate emotional distress and reduce the psychological burden of MS. This highlights the importance of narrative therapy, peer support and resilience-building interventions to promote healthy adjustment in illness.

### 3.2. How Stigma Disrupts Identity Formation

Identity construction in the context of chronic illness is a recursive, complicated, and socially motivated process. Faced with stigma—whether expressed through overt discrimination, subtle microaggressions, or perceived social devaluation—individuals are compelled to negotiate a challenging and often precarious landscape of identity [57]. This negotiation is frequently marked by tension between the public self (how one is seen) and the private self (how one experiences the illness internally), resulting in fragmented or contradictory identity positions [27]. Grytten and Måseide [27] notably observed that PwMS often experience a sense of embodied illegitimacy, i.e., the feeling that their illness, particularly when symptoms are invisible, is not viewed as valid or credible by others. This perceived lack of legitimacy undermines identity coherence and impedes the validation of the illness experience. Research consistently shows that the invisibility of an illness exacerbates this tension. Individuals with chronic but invisible illnesses are more likely to experience a heightened illness self-concept shaped by internalized and anticipated stigma, leading to question legitimacy and struggle with identity integration [58]. A central element in this identity negotiation is the decision to disclose or conceal one’s illness. While concealment may offer short-term protection from external judgment, it often leads to increased psychological distress and hinders identity development [59]. In occupational settings, perceived identity threat—especially when individual expects negative treatment due to an invisible illness—can contribute to heightened psychological distress and reduced performance, particularly if the illness is not outwardly visible [60]. This pressure contributes to a fractured self-narrative, as individuals must balance the competing demands of appearing “normal” while coping with an often-invisible burden. Furthermore, internalized stigma has been shown to diminish feelings of self-determination and hope, further complicating identity work and delaying psychological adjustment [61]. The internal conflict of presenting oneself as healthy while being sick, creates a dissonance that many attempt to resolve by constructing a coherent self-narrative. In sum, the stigma of chronic illness—especially when tied to the invisibility of illness—has a destabilizing effect on identity. While concealment may provide short-term protection from social judgment, it ultimately impairs identity integration, self-acceptance, and psychological well-being over time.

### 3.3. Narrative Identity and the Re-Authoring of the Self

As already mentioned, one effective method of resisting the corrosive effects of stigmatization on identity in chronic illness is narrative. In particular, the so-called narrative identity theory states that people construct internalized, evolving life stories that give them a sense of unity and meaning [62]. Narratives are important because they provide a symbolic structure through which individuals can reconstruct their disrupted lives, reinterpret suffering, and transform the illness experience from a source of shame to a place of meaning-making. Narrative identity theory [62] also explains how people develop internalized, evolving life stories that give unity and meaning to their lives. In the context of MS, such narratives can either be constrained by stigma or altered through conscious meaning-making and social validation. In the context of chronic diseases such as MS, these life stories are disrupted and often trigger a biographical rupture in which the individual struggles to reconcile their former, healthy self with the limitations and uncertainty caused by the illness. Narrative reconstruction becomes essential to repairing the self and allows for continuity despite change. Indeed, studies have shown that people who embrace meaningful narratives, e.g., stories of resilience, transformation or advocacy, are more likely to demonstrate psychological well-being and adaptive coping strategies [63,64]. The presence of stigma, especially if it has been internalized, can severely hinder narrative coherence. Studies have shown that people who perceive or internalize stigma often experience chaotic or fragmented illness narratives, reflecting emotional distress, unresolved identity tensions and a lack of psychological integration and often correlate with increased depression, anxiety and low self-esteem [19,58]. Fragmented stories reflect not only the psychological toll of the illness, but also the inability to find a coherent identity position between “sick” and “normal”. In contrast, people who construct meaningful narratives—those that emphasize resilience, self-determination or personal growth—are more likely to report positive psychological outcomes and adaptive coping strategies. Participating in practices such as diary writing, blogging, peer group storytelling and artistic expression have been shown to facilitate the narrative reconstruction [65]. For example, creative writing and personal storytelling have been found to enable people with mental illness to counteract internalized stigma and develop a new sense of self-worth [66]. The literature shows that people who engage in this type of storytelling are better able to maintain both meaningful relationships and a sense of self-continuity [67,68]. Resilience in narrative identity work should not be underestimated. Acting as both mediator and moderator between stigma and identity outcomes, resilience helps individuals transform adversity into empowering life stories. For example, studies on inflammatory bowel disease demonstrate that resilience mitigates the negative effects of stigma on illness identity, leading to positive identity integration and enhancing psychological well-being [19]. In summary, storytelling is not merely a therapeutic intervention, it is a fundamental identity process. Through storytelling, individuals reclaim authorship over their lives, resist stigma, and reconstruct a self that is coherent, empowered, and socially affirmed.

### 3.4. Empirical Evidence on Stigma and Illness Identity in MS

Quantitative studies show a strong correlation between stigma and maladaptive disease identity in PwMS. Stigma, particularly internalized stigma, has a strong influence on how people define themselves in relation to their disease. A seminal study by Stepleman et al. [18], which used different tools to measure perceived stigma and psychological well-being, demonstrated that perceived stigma correlated positively with engulfment and negatively with acceptance and enrichment. These findings illustrate how internalization of the pejorative stigma of social alienation distorts self-perception and shifts from healthy integration to a self-concept characterized by psychological over-identification with the disease and inadequacy. Further evidence comes from a number of studies using the Neuro-QoL Stigma subscale, including that of Glanz et al. [69], which indicate that higher stigma scores correlate significantly with lower self-efficacy, lower sense of purpose and lower sense of control, psychosocial resources necessary for productive development. These correlations indicate that stigma is both a psychosocial stressor and an emergent developmental force that affects how people portray and organize their illness experiences. From a clinical perspective, these findings have an important implication. Indeed, reducing stigma could have a dual purpose: not only alleviating suffering, but also helping to build a more adaptive illness identity. An accepting or enriching illness identity correlates with increased treatment adherence, psychological flexibility and social integration [70], while engulfment and rejection correlate with suffering, social withdrawal and identity diffusion [71,72]. This has implications for individual treatment and the design of psychosocial interventions. The stigma-identity dynamic does not exist in a cultural and social vacuum, but is strongly influenced by cultural values and social norms. In collectivist cultures, for example, an illness may be seen not only as a personal problem, but also as a source of shame or a burden on the family. This cultural script can facilitate concealment, making seeking help more difficult and creating a rejection-oriented identity in which people deny their illness so as not to disrupt social harmony [73,74]. Therefore, in developing approaches to reduce stigma, it is important to understand both psychological and cultural dynamics. Universal models based on one-size-fits-all approaches are unlikely to be effective unless they take into account the different ways in which identity, self-worth and social belonging are constructed in a particular context [75]. For example, open disclosure and empowerment-based interventions that might be used in individualistic cultures would need to be reframed in a more collectivistic context with a focus on community unity, the role of the family and relational security [76]. Clinicians must also be aware of the interactions between stigma, gender, age, ethnicity and sexual identity, as these can exacerbate the nexus between illness and identity. In conclusion, the relationship between stigma and disease identity in MS is dynamic, multilayered, and socially embedded. It not only shapes how individuals perceive their illness, but also how they define themselves in relation to it, and, ultimately, how they cope. Future studies should examine these relationships using longitudinal studies and ethnically and culturally diverse samples to develop interventions that lead to identity resilience and psychological development.

### 3.5. Psychological Interventions to Reduce Stigma and Restore Identity: Current Practices and Future Directions

Traditional approaches to the treatment of MS have focused predominantly on the neurological symptoms and disease-modifying therapies, but it is important to consider the significant psychosocial burden, particularly the pervasive effects of stigma. In general, identity-forming mechanisms are strongly influenced by internalized stigma, societal discourse, and broader cultural narratives. Interventions that target these narrative and cognitive processes can help to facilitate the formation of a more adaptive illness identity. In this regard, there is growing evidence to support the use of evidence-based psychological interventions to counter internalized stigma and mitigate the emotional and cognitive costs of MS. One of the best supported methods is cognitive behavioral therapy (CBT), which focuses on self-stigma and self-esteem. This approach has been shown to be effective in improving mood and reducing self-stigmatizing thoughts in other marginalized populations [44,45]. On the other hand, group CBT, especially when based on social identity theory, promotes shared understanding and coping, but carries risks in situations of high identity threat and low group coherence [77]. Acceptance and commitment therapy (ACT) has also shown promise in supporting PwMS. Through its core processes, i.e., cognitive defusion, values-based action, and cultivation of the self as context, ACT enables people to distance themselves from stigmatizing thoughts while continuing to participate meaningfully in life [78]. In line with ACT theory, narrative enhancement and cognitive therapy (NECT) has been shown to be effective in reconstructing a coherent sense of self. NECT was originally developed for people with severe mental illness and combines psychoeducation, cognitive restructuring and narrative intervention to reduce self-stigma and support identity recovery [61,79,80,81]. A quasi-experimental study found that participants who took part in NECT experienced a significant reduction in self-stigma as well as an increase in self-esteem, hope and quality of life [82,83]. More recently, pilot studies in countries such as the Netherlands have begun to validate the feasibility and efficacy of NECT in a variety of clinical settings [81]. Crucially, NECT is also suitable for people with intersectional stigma. A qualitative study with LGBTQ+ participants illustrated how “double stigma”, i.e., both the stigma resulting from mental illness and the stigma of marginalized sexual/gender identity, can affect recovery trajectories, influence self-concept, and impede disclosure [37,84]. Adapting NECT for PwMS may offer promising ways to reframe illness experiences and support psychological integration. Among the various psychological approaches explored in the literature on MS, mindfulness-based interventions have shown particular promise in addressing both the psychological correlates and internalization of stigma in PwMS. A systematic review has shown that such interventions consistently improve quality of life, symptoms of depression and anxiety, and emotion regulation in PwMS [85]. Beyond these general benefits, mindfulness appears to have a targeted effect on self-stigma. Specifically, empirical studies have shown that mindfulness-based programs can attenuate internalized stigma by promoting important psychological processes such as decentering, increased self-compassion, and improved self-efficacy [86,87,88]. These mechanisms can empower people to disengage from stigmatizing self-narratives and develop a more accepting, resilient relationship with themselves. These elements are particularly valuable in the context of chronic illness, where identity and self-worth are often challenged. While CBT, ACT, NECT, and mindfulness-based therapies have received considerable empirical attention in terms of reducing stigma in people with disabilities, there are a number of models in the literature that have been shown to reduce internalized stigma and restore identity coherence in stigmatized populations. Among them, compassion-focused therapy (CFT) aims to address shame and self-criticism, which are at the core of internalized stigma. CFT focuses on activating the affiliative emotional system to develop self-compassion and attenuate self-stigma, especially in people with early relational trauma or chronic feelings of inadequacy [89]. CFT has been shown to significantly improve cognitive–emotional regulation in clinical populations, including chronically ill patients. For example, in people with cardiovascular disease, CFT led to a significant improvement in exercise tolerance and a reduction in difficulties with emotion regulation [90]. Another useful approach for improving the quality of life of people with disabilities is interpersonal therapy (IPT) [91]. Originally developed for the treatment of depression, it targets role transitions, interpersonal conflict and grief—emotional challenges commonly encountered during adjustment to MS. IPT has been shown to be highly effective in treating social withdrawal and isolation due to stigma, particularly by alleviating relationship difficulties that often follow the onset of chronic illness [91]. In PwMS, IPT can restore interpersonal identity and promote reintegration into the community, especially for those who need to adapt to relational conflict or loss of social roles. Psychoeducation and coping programs—although often underrepresented in the stigma literature—are widely used in various clinical contexts [92,93,94], including in digital form [95]. These approaches can be instrumental in promoting an accurate understanding of illness, validating emotional responses [96] and redefining stigma as an expression of societal prejudice rather than individual failure [97]. Furthermore, there is evidence that these types of programs have been successful in reducing internalized stigma in patients with mental disorders by improving understanding of their illness and adaptive attributional styles [98]. Therefore, these approaches could be adapted in people with disabilities to explore the impact of identity and the triggers of stigma, particularly in the context of transition to illness or diagnosis. In addition, group-based stigma-reduction programs, while less mature in MS, have had considerable success in similar populations such as HIV and schizophrenia. For example, the Honest, Open, Proud (HOP) intervention, which targets stigma-related disclosure issues, has been shown to be extremely helpful in alleviating stigma stress and increasing empowerment [99,100]. In addition, HOP helps participants to make well-informed decisions about disclosure and restore their self-concept through guided group work and support. As most PwMS struggle with invisible signs and conflicts related to identity, peer-led formats provide an explicitly validating environment for emotional release and the restoration of personal control [101]. Expressive therapies and creative arts, including digital storytelling, illness memoir writing, and therapeutic photography, also provide symbolic and nonverbal avenues for reconstructing disrupted personal narratives. The flexibility of expressive therapies and creative arts makes them particularly appropriate for individuals with cognitive fatigue or verbal processing difficulties [102], symptoms common in MS. Finally, it is important to point out that the critical factor in the success of any therapy is the therapeutic alliance. Indeed, there is consistent evidence that the quality of the therapeutic alliance is related to the success of therapeutic treatment, across a wide range of patient types, treatment modalities used, antecedent problems, contexts and measurements [103,104,105,106]. In summary, while CBT, ACT, and mindfulness-based interventions remain at the center of the evidence base for stigma treatment, a broader range of approaches (i.e., CFT, IPT, psychoeducation, NECT, peer-led interventions, expressive therapy, psychoanalysis) may offer unique and, importantly, complementary benefits. In summary, improving the well-being in PwMS requires a nuanced, multi-faceted approach that goes beyond a single therapeutic model. CBT, ACT and mindfulness remain foundational, but the integration of narrative, interpersonal, compassion-focused and psychoeducational strategies—alongside peer-led and expressive modalities—enriches the therapeutic landscape. By embracing this diversity, healthcare professionals can more effectively support identity reconstruction, emotional resilience and social reintegration in PwMS navigating the complex psychosocial reality of MS.

## 4. Future Research Directions and Conclusions

Although the literature on stigma and psychological adjustment in PwMS is growing, there are a number of critical gaps. Of greatest importance is the longitudinal research that looks at how stigma changes over the course of the disease—at stages such as diagnosis, transition to treatment, relapse, and significant life events—is lacking. Clarifying how people move through their illness identity—from one of rejection or engulfment to one of acceptance or affirmation—could identify pathways to resilience and provide direction for stage- and identity-specific interventions [17]. Despite the increasing recognition of the impact of stigma, there is a lack of intervention studies. Interventions such as ACT, NECT and peer-led groups have been shown to be useful in reducing self-stigma and improving mental health in groups with HIV, psychosis and epilepsy, but are not well enough studied in PwMS [79,100]. Mixed methods studies combining established scales (e.g., SSCI-8, Neuro-QoL, Reece Stigma Scale) with qualitative interviews could provide greater insight into the lived experience of stigma and how an intervention promotes significant psychological change. Furthermore, cultural adaptation of stigma instruments is warranted. Soltani et al. [32] highlight that Western instruments are potentially blind to the culturally ingrained forms of stigma in non-Western groups where membership obligations, shame, and social judgment significantly influence disclosure and illness-identity decisions [32]. Validation and adaptation in a variety of cultural and linguistic contexts will make the tools more useful and equitable for global MS care. To move forward in this area, interdisciplinary collaboration is essential, with neurologists, psychologists, sociologists and PwMS advocates working together to develop person-centered models of care that take into account the complex biopsychosocial nature of MS. Crucially, further work will also take into account technological advances to deliver interventions in scalable, accessible formats, including for people with limiting fatigue or mobility issues [102]. Most critically, stigma in MS must be tackled by more than simply targeting cognitive distortions alone; it requires an integrative approach capable of addressing the embodied, relational, and identity-disruptive nature of stigmatization. In this, we propose the development of a mindfulness stigma reduction program for PwMS, grounded in theoretical and empirical underpinnings. Besides their cognitive benefits, MBIs increase a non-judgmental perception of bodily sensations, promote emotional regulation, and promote self-compassion, factors that are most important for reducing internalized stigma, particularly when such stigma is manifest through body shame, social isolation, and a disjointed sense of illness identity. These processes align with theoretical models, such as stigma-related cognitive fusion, avoidance, and self-discrepancy [107]. Group-delivered MBIs can also offer corrective interpersonal experiences, through mutual stories and recognition of shared humanity, and thereby enhance identity coherence and social connectedness. Although MBIs have shown preliminary efficacy in improving mood, reducing fatigue, and enhancing self-efficacy in PwMS, later iterations should specifically incorporate stigma-reduction goals, narrative self-reconstruction, and cultural adaptation. These interventions would be augmented by brief formats, accessible delivery (e.g., online or app delivery), and peer co-facilitation. For what has been said so far, a combined model of stigma-awareness and mindfulness could be key to restoring dignity, agency, and mental well-being in PwMS, becoming a fundamental component of holistic MS care. Aside from its clinical importance, stigma research in individuals with multiple sclerosis provides unique theoretical insights into the general study of social stigmatization. In contrast to the usual examined populations (e.g., mental illness, HIV), PwMS frequently endure a fluctuating, partially hidden condition characterized by uncertainty, progressive deterioration, and socially unclear symptoms. These characteristics defy linear stigma models and require a more dynamic conceptualization incorporating temporal instability and identity fluidity. The recursive interaction among perceived, anticipated, and internalized stigma and illness-identity construction in PwMS also shows how stigma is not just a social judgment but an ongoing cognitive-affective process influenced by meaning-making and self-concept. Furthermore, the MS case also complicates traditional stigma theory by highlighting the tension of concealability and legitimacy, two dimensions most relevant for diseases without visible markers. In this way, the stigmatization of PwMS can be regarded as a paradigmatic window for the elaboration of stigma theory where chronicity, invisibility, and self-narrative intersect. Theoretical frameworks in the future would do well to include these dimensions to more effectively encapsulate the lived intricacies of stigma in chronic, neuroprogressive disease. In conclusion, stigma in PwMS is a primary concern, a main determinant of emotional well-being, identity consistency and engagement with care. The effects are cumulative, recursive and often imperceptible. A stigma-sensitive, narrative-based and culturally competent model of care needs to be integrated into mainstream MS care, not just to cope with the condition, but also to enable wellness and identity rehabilitation for people with chronic, socially stigmatizing disease.

## Abbreviations

The following abbreviations are used in this manuscript:

MS	Multiple sclerosis
PwMS	People with multiple sclerosis
ACT	Acceptance and commitment therapy
CBT	Cognitive behavior therapy
NECT	Narrative enhancement and cognitive therapy
MBIs	Mindfulness-based interventions
CFT	Compassion-focused therapy
IPT	Interpersonal therapy
HOP	Honest, Open, Proud
